# Characterization and Preliminary Application of Phage Isolated From *Listeria monocytogenes*

**DOI:** 10.3389/fvets.2022.946814

**Published:** 2022-07-20

**Authors:** Tianhao Li, Xuehui Zhao, Xuejian Wang, Zijian Wang, Changqing Tian, Wenjing Shi, Yumei Qi, Huilin Wei, Chen Song, Huiwen Xue, Huitian Gou

**Affiliations:** ^1^College of Veterinary Medical, Gansu Agricultural University, Lanzhou, China; ^2^Infectious Diseases Section, Xigu District Animal Disease Prevention and Control Center, Lanzhou, China; ^3^Infectious Diseases Section, Gansu Province Animal Disease Prevention and Control Center, Lanzhou, China

**Keywords:** *Listeria monocytogenes*, Phage, Isolation, characterization, combination therapy

## Abstract

*Listeria monocytogenes* (LM) is one of the four major foodborne bacteria that cause bacteremia and meningitis. To explore the control of listeriosis with natural phages, we used the double-layer agar plate method to isolate LM from slaughterhouse sewage and designated LP8. The result of electron microscopy indicated that the phage belonged to the family of *Myoviridae*. Whole-genome sequencing indicated that the genome size of LP8 is 87,038 bp and contains 120 genes. Mice were infected with LM and treated with penicillin G sodium, LP8, and the combination of these two. From the levels of lymphocyte subsets (CD^4+^, CD^8+^), the expression of cytokines (TNF-α, IL1β, IL-10, and IFN-γ), observation of pathological changes in organs (heart, liver, spleen, kidney, and brain), and the bacterial load of the spleen, we concluded the therapeutic effect of LP8 against listeriosis and demonstrate the feasibility of a combined therapy to reduce the use of antibiotics. This provides a new avenue for the treatment of listeriosis.

## Highlights

- A Listeria monocytogenes phage LP8 was isolated from slaughterhouse sewage. It was DNA virus, genome size was 87,038 bp, coded 120 genes and belong to Myoviridae.
- The combination of LP8 and antibiotics can make up for the influence of the biological activity of the phage itself on the treatment. Meanwhile, the addition of phage can reduce the use of antibiotics and achieve the effect of reducing and limiting resistance.

## Introduction

*Listeria monocytogenes* (LM) is one of the four major foodborne pathogens that cause bacteremia and meningitis. LM can survive under various stress conditions, including extreme temperature, drought, low pH, and high salinity (1–4). In recent years, Listeria has been shown to include 27 species. However, only two strains, *L. monocytogenes* and *L. ivanovii*, can cause human and animal diseases (5). Hence, the risk of infection from contaminated food increases after refrigeration (6). About 20 to 65% of the deaths from foodborne infections are caused by LM in food hygiene events in the United States (7–9). In 2018, the fatality rate of listeriosis among detected cases in EU countries was 15.6% (10). It is generally accepted that 99% of infections caused by LM are non-invasive infections through the fecal-oral route, secondary to invasive infections (11). The diseases caused by LM are predominantly treated with drugs, such as penicillin, fluoroquinolones, cephalosporins, and vancomycin (12). LM carried the *plcb* and *iap* genes, which make the bacterium highly resistant to drugs, such as penicillin and erythromycin (13). *L. monocytogenes* can cross the intestinal barrier by the active invasion of host cells, which is mainly triggered by internalins InlA and InlB. Other virulence factors, such as listeriolysin O (LLO) and phospholipases are activated by the low pH inside the phagolysosome and allow the bacteria to escape into the cytoplasm (14). After the release into the host cell cytoplasm, *L. monocytogenes* can multiply and spread to neighboring cells, driven by ActA-mediated polymerization of host-cell actin (15). This strategy enables the pathogen to cross host tissue barriers, such as the placental and the blood-brain barrier.

Bacteriophages (phage) are viruses that infect bacterial hosts but are not harmful to eukaryotic organisms. Phages cannot replicate independently of their bacterial hosts. Their host ranges are determined by various factors, including the specificity of the phages' host-binding protein and the host's resistance (16). In recent years, phages have been used as alternatives to antibiotics indicating large potential. However, the high specificity for host bacteria and sensitivity to environmental factors (temperature, pH, etc.) might be limited to the use of phages on a large scale. Therefore, cocktail, phage transformation, and other methods have been proven to be effective measures to overcome the above shortcomings (17). In recent years, to face the emerging drug resistance problem of *Listeria monocytogenes* the use of phages to treat listeriosis will become a feasible approach.

In this study, an LM phage was isolated from slaughterhouse sewage. The morphology, biological characteristics, and whole-genome sequence of the phage were analyzed. To assess the effects of the phage intervention, a mouse model of LM infection was established and treated with the phage, antibiotic, or a phage–antibiotic mixture, as well as comprehensive analysis of lymphocyte subsets, cytokines, pathological changes, and bacterial load.

## Materials and Methods

### Mouse Experiments and Ethics

Female 6∽8-week-old Kunming mice (SPF Standard Animal Laboratory, Lanzhou University Medical Laboratory Center, Lanzhou, China) were housed in a vector-free animal facility, under controlled environmental conditions with a 12-h day-night cycle, and free access to food and water. All animals followed the relevant protocols of animal welfare of the Gansu agricultural university college of veterinary medicine (Lanzhou, China).

Kunming mice are the outbred mice with the largest production and usage in China. The gene pool is large, the gene heterozygosity rate is high, and it is characterized by strong disease resistance and adaptability, as well as high reproduction and survival rates.

### Strains

Among the tested 50 *Listeria* strains, four standard strains (NCTC 19890, ATCC 19112, ATCC 19111, and ATCC 19115) and 46 wild strains (18 strains of *L. monocytogenes*, 10 strains of *L. welshimeri*, and 22 strains of *L. innocua*) were all wild strains isolated from beef and mutton samples in parts of Gansu province in previous work in this laboratory. After programmed thawing, they were used to inoculate into tryptic soy broth supplemented with yeast extract (TSB-YE; Solarbio, Beijing, China) and cultured overnight at 37°C with shaking at 220 pm.

### Phage Isolation and Characterization

The sewage was centrifuged at 3,000 × g for 30 min at 4°C, and filtered through a.22 μm filter. The double-layer agar plate method was used for phage separation (18). A plaque was aspirated into a 1.5 mL sterile centrifuge tube containing 500 μL of magnesium sulfate buffer (Solarbio, Beijing, China), and lysed statically for 4 h at 4°C. The lysate was incubated on double-layer agar plates. The above steps were repeated 5 6 times to purify the phage.

The bacterial culture fluid (100 μL) and lysate (100 μL) were added to 100 mL of TSB-YE, and the mixture was cultured overnight at 37°C with shaking. Polyethylene glycol 8,000 (Solarbio, Beijing, China) was used to prepare a high-titer phage solution. To determine the phage host spectrum, the phage was separately cultured with 50 strains of *Listeria* spp, the plaque-forming units (pfu) were counted, and the electroplating efficiency (EOP) was calculated as follows: EOP = phage titer in the test bacteria/phage titer in the host bacteria(Host bacteria is the bacteria in which the phage was originally isolated. Test bacteria is the bacteria used to test excepted host bacteria) (19). The phage solution was stained with phosphotungstic acid and observed with transmission electron microscopy (Hitachi HT 7,700, Hitachi Ltd, Tokyo, Japan).

Different phage/bacterium ratios were tested to determine the optimal multiplicity of infection of phage (MOI = 0.001, 0.01, 0.1, 1, 10, or 100). Under the optimal conditions for infection, bacterial and phage cultures were subjected to gradients of temperature (25°C, 35°C, 45°C, 55°C, 65°C, and 75°C) and pH (2, 4, 6, 8, 10, and 12), and different solvents (methanol, ethanol, glycerol, isoamyl alcohol, magnesium sulfate buffer, and chloroform) to determine optimal growth conditions, and their growth was measured per 10 min to 2 h. The data were used to construct one-step growth curves.

### Phage Genome-Wide Analysis

The genome was extracted from the phage with the Tris-HCL method (20). The genome was digested with DNase I, RNase I, and mung bean nuclease (Solarbio) to determine the genome type. The phage genome was scanned and sequenced with Illumina sequencing technology. The databases RefSeq nonredundant (Nr) protein (RefSeq: NCBI Reference Sequence Database, nih.gov), Clusters of Orthologous Groups of proteins (COG, https://www.ncbi.nlm.nih.gov/research/cog), Kyoto Encyclopedia of Genes and Genomes (KEGG, https://www.kegg.jp/kegg/), Swiss-Prot (www.sib.swiss/swiss-prot), and Gene Ontology (http://www.geneontology.org/GO.indices.html) were used to annotate the genes. Finally, the SnapGene software (Insightful Science; available at snapgene.com) was used to analyze the distributions and functions of the genes.

### Listeriosis Infection Model and Phage Intervention

Sixty female Kunming mice were randomly divided into a blank control group, toxic reaction group, antibiotic treatment group, phage treatment group, and combined treatment group (Appendix 1). About 100 μL bacterial culture with OD_600_ = 0.6 (which is equivalent to a bacterial concentration of 2.09 × 10^10^CFU/mL, Appendix 2) was injected into each mouse. The phage injection was converted according to the detected optimal MOI (bacterial concentration × MOI), and the injection volume was 100 μL. Among the six antimicrobial drugs, ATCC19111 had the highest resistance to penicillin G sodium and was sensitive to other antibiotics. In addition, among many antibiotics, β-lactam antibiotics are the most widely used. Finally, penicillin G sodium was selected as the tested antibiotic (Appendix 3). According to the *in vitro* plate bacteriostatic test, the minimum inhibitory concentration of single penicillin G sodium is 11 mg/L (Appendix 4), and the minimum inhibitory concentration of the combination with phage LP8 is 7 mg/L (Appendix 5).

### Sample Detection

Peripheral blood (100 μL) was collected from each mouse and 5 μL of allophycocyanin (APC)-conjugated anti-mouse CD^3+^ antibody **(0.2** mg/mL), 2 μL of phycoerythrin (PE)-conjugated anti-mouse CD^4+^ antibody **(0.5** mg/mL), or 5 μL of fluorescein isothiocyanate (FITC)-conjugated anti-mouse CD^8+^ antibody **(0.2** mg/mL) (BioLegend, San Diego, CA, USA) were added to the sample to stain. All samples from each group were analyzed with flow cytometry. Treestar FlowJo v10.0 was used to analyze the data.

Mouse serum was collected to test the expression of cytokines, tumor necrosis factor α (TNF-α), interleukin 1β (IL-1β), interleukin 10(IL-10), and interferon γ (IFN-γ). ELISAs kit (Jiangsu ELISA Co., Ltd, Jiangsu, China) was used to test the above value and detailed operation according to kit instructions.

Heart, liver, spleen, kidney, and brain tissues were sampled from each mouse and steeped in 4% formaldehyde. Sections (3 μm) were cut, stained with HE, and observed microscopically at 400× magnification (Olympus Corporation, Japan). Sections were also stained with Giemsa and observed at 1,000× magnification.

The spleens were collected aseptically from each group of mice and pooled according to the group in sterile 1.5-mL centrifuge tubes. Sterilized stainless-steel balls and 1 mL of phosphate-buffered saline (PBS) were added, and the tissues were ground for 300 s with a high-throughput tissue grinder (Ningbo Biotechnology Co., Ltd, Zhejiang, China). Each spleen suspension was diluted with PBS (10^−1^, 10^−3^, 10^−5^, 10^−7^, and 10^−9^), and an aliquot (100 μL) of each dilution was added to a solid TSB-YE medium, incubated at 37°C for 12 h, and colonies were counted. Five biological replicates were analyzed per sample.

### Data Analysis

All comparisons among groups were analyzed with an independent- samples *t-test* and analysis of variance (ANOVA), with the SPSS software. The data were analyzed and visualized with the Origin software (Origin Lab Corporation Northampton, MA, USA).

## Results

### Isolation and Identification of Phage

**Figure 1:**
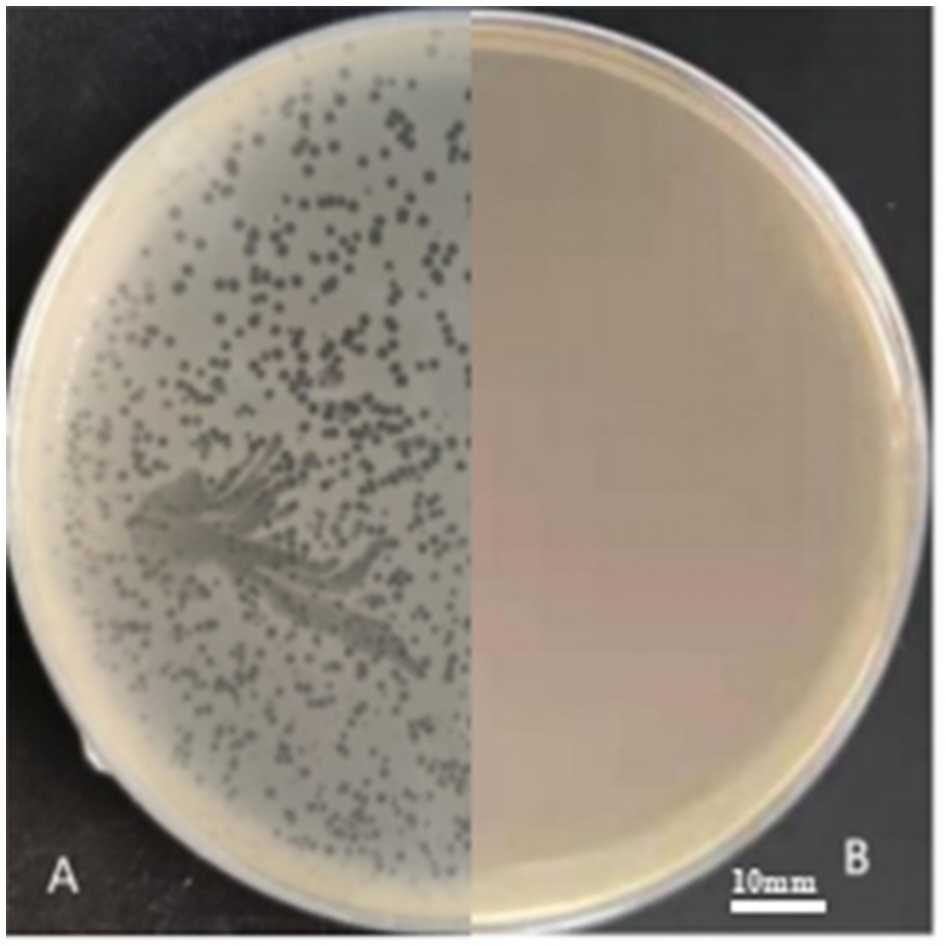
LP8 plaques observed on plate. The host strain was ATCC19111 (1/2a). The plaques, approximately 2 mm in diameter, were seen on the plate. **(A)** is separation plate. **(B)** is blank plate.

**Figure 2:**
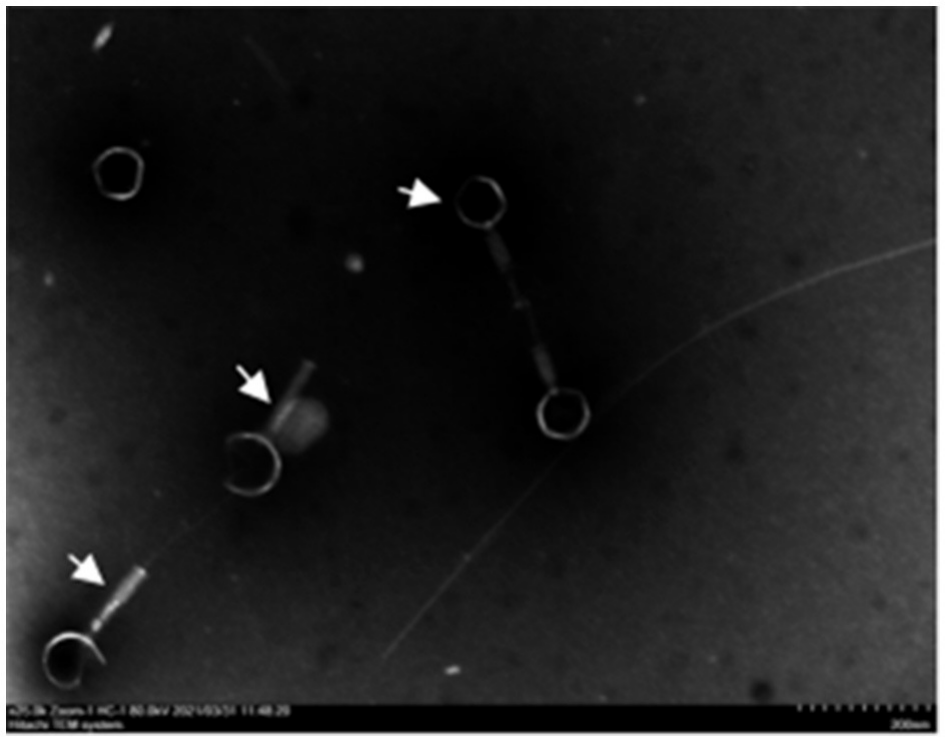
Transmission electron microscopy (TEM) image of LP8. White arrows in the figure indicate phage. The phage has a hollow needle-like structure; the tail consists of an envelope and the base consists of a tail needle. The average diameter of the phage head is 80 nm and the average length of the tail is 120 nm. Scale bar: 200 nm.

In three slaughterhouse sewage samples, a total of four phages were isolated and named LP1, LP2, LP3, and LP4. After purification, it was found that LP1 and LP2 were not *Listeria monocytogenes* phages, while LP3 and LP4 were found to be the same phage by whole genome sequencing. To distinguish, LP3 and LP4 are uniformly named LP8 for subsequent experiments (Figure 1). Host spectrum showed that LP8 lysed 18 strains of *L. monocytogenes* and 5 strains of *L. welshimeri* (Appendix 6). Transmission electron microscopy showed that LP8 belongs to *Myoviridae* (Figure 2). The optimal MOI for LP8 was 1, the optimal growth temperature was 45°C, the optimal pH was 7, and isopentyl alcohol could lead to the LP8 inactivity (Appendix 7).

### Phage Genome Analysis

**Figure 3:**
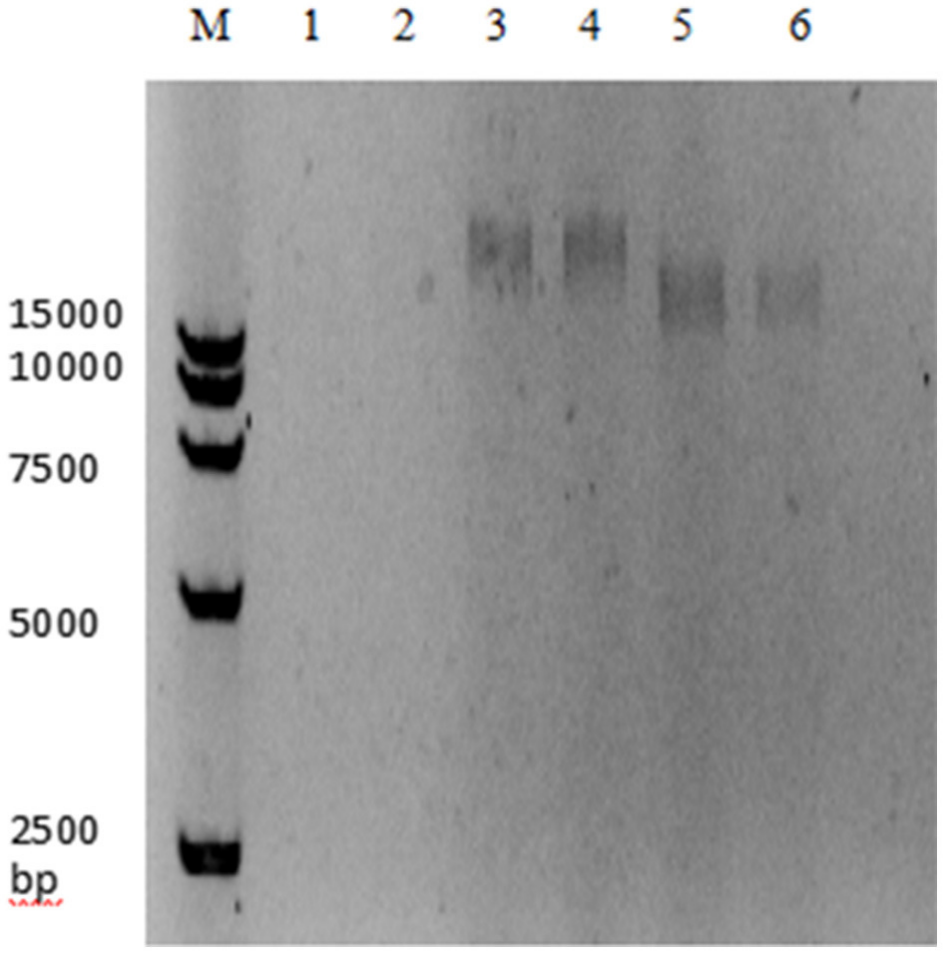
Digestion electrophoresis of LP8 phage genome. M, DNA Marker 15,000;1, 2, DNase;3, 4, RNase;5, 6, Mung bean nuclease.

**Figure 4:**
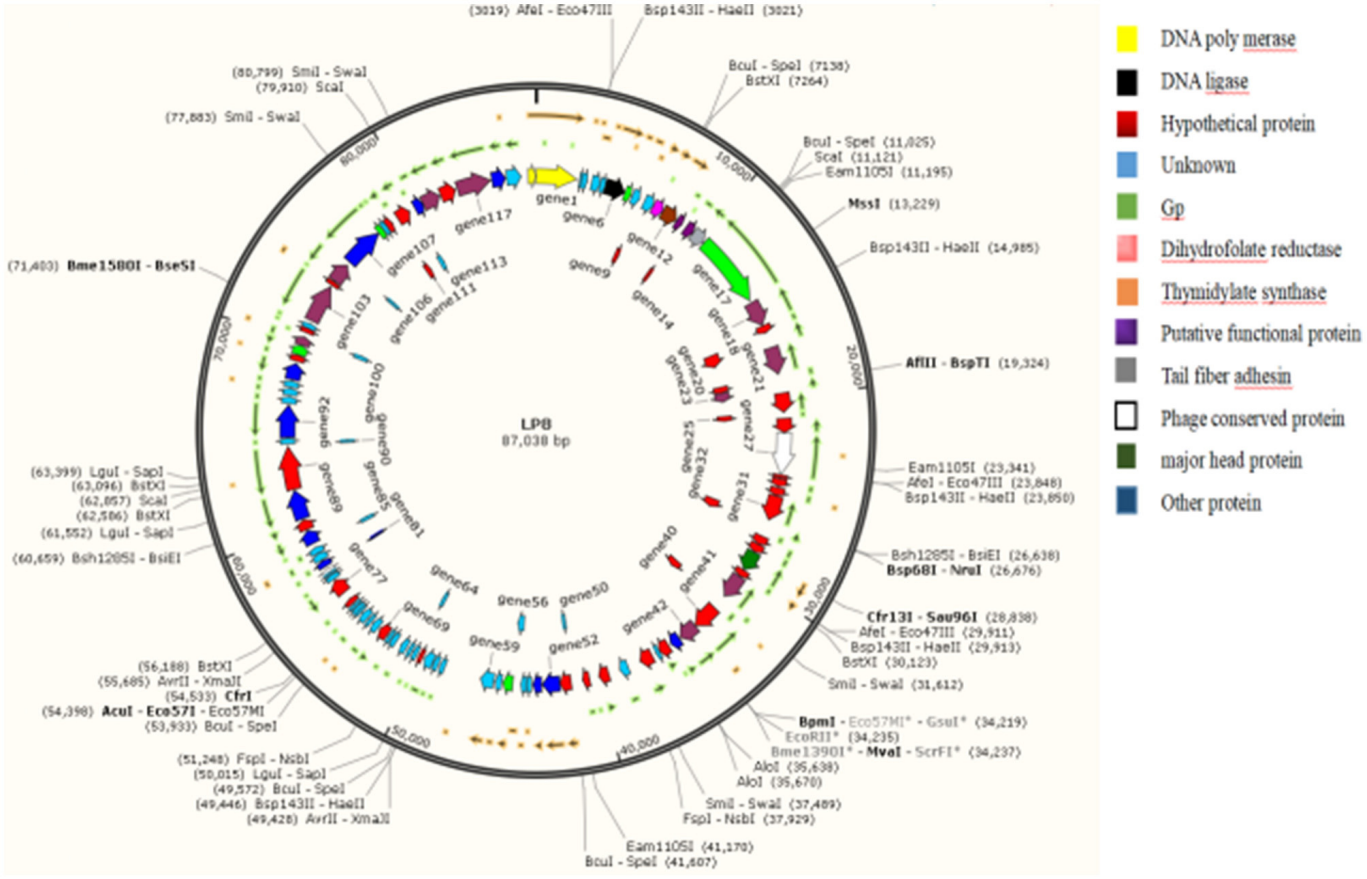
Whole-genome map of LP8. Length of the phage LP8 genome is 87,038 bp, with a total of 120 coding genes. Length of the coding region accounts for 87.69% of the genome length. Six genes encode proteins involved in phage synthesis, 20 genes encode proteins involved in phage-related biological processes, and 11 genes encode proteins involved in molecular functions such as binding or catalysis.

The genome of the LP8 phage was double-stranded DNA (Figure 3). Whole-genome sequencing indicated that the genome size of LP8 was 87,038 bp and encoded 120 genes. These genes encoded proteins involved in phage synthesis, invasion, assembly, signal transduction, and related functions (Figure 4). The non-structural proteins were predominantly involved in regulatory roles and signal transduction. The phage sequence was uploaded to the NCBI database (GenBank: OK283618.1).

### Evaluation of Therapeutic Effects of LP8

**Figure 5:**
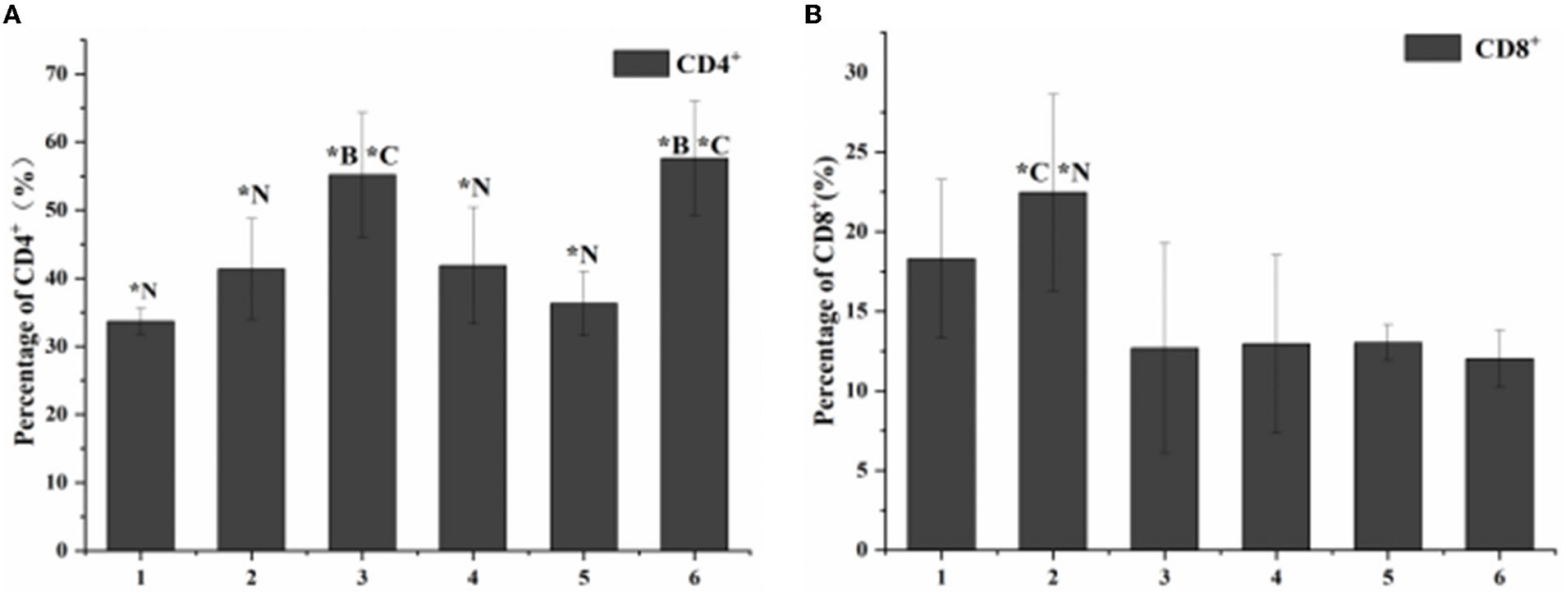
Statistical chart of the proportion of lymphocyte subsets. 1. Blank control group. 2. Toxic group. 3. Penicillin G sodium treatment group. 4. Phage therapy group. 5. Combination therapy group. 6. Non-intervention group. The values are means and standard deviations (SD) (*n* = 5). *C < 0.05. Each group vs. combination therapy group. *B **<** 0.05. Each group vs. blank control group. *N **<** 0.05. Each group vs. non-intervention group.

According to the results of flow cytometry, the proportion of CD^4+^ cells in the blank control group was 34%, while the proportion of CD^8+^ cells was 16%. The proportion of CD^4+^ and CD^8+^cells in the toxicity group was 41 and 25%. The proportion of CD^4+^ and CD^8+^cells in the antibiotic treatment group was 56 and 12%. The proportion of CD^4+^ and CD^8+^cells in the phage-treated group was 42 and 13%, respectively. The proportion of CD^4+^ and CD^8+^ cells in the combined treatment group were 37 and 16%. The proportion of CD^4+^ and CD^8+^cells in the non-intervention group was 58 and 11% (Figure 5).

**Figure 6:**
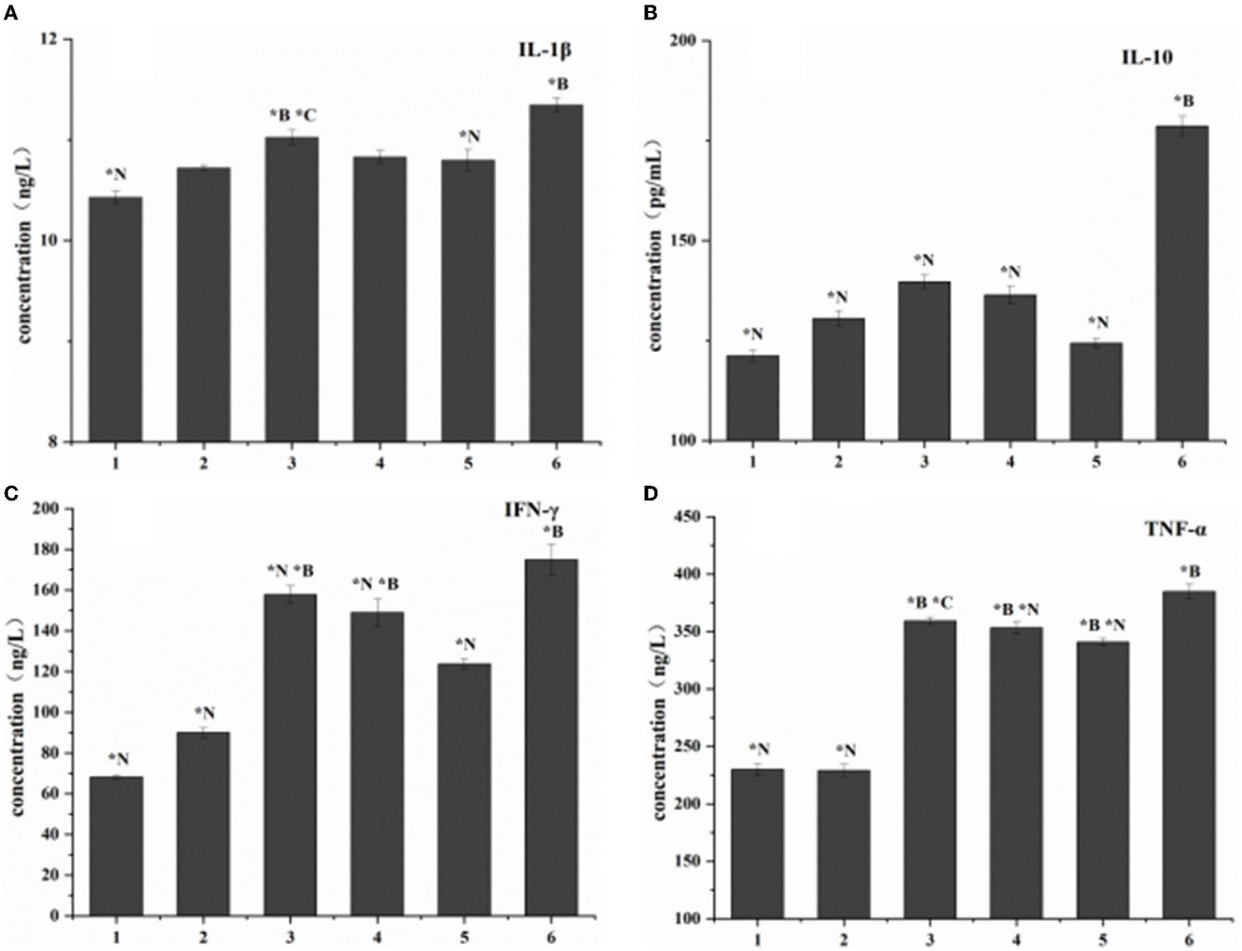
Cytokine expression in listeriosis model. 1. Blank control group. 2. Toxic group. 3. Penicillin G sodium treatment group. 4. Phage therapy group. 5. Combination therapy group. 6. Non-intervention group. The values are means and standard deviations (SD) (*n* = 5). *C<0.05. Each group vs. combination therapy group. *B < 0.05. Each group vs. blank control group. *N<0.05. Each group vs. non-intervention group.

Compared with the blank control group, the expression levels of the four cytokines (TNF-α, IL-1β, IL-10, and IFN-γ) increased *in vivo* in all treatment groups. The no-intervention group had the highest expression levels. Among the three treatment groups, the cytokine expression in the combined treatment group had the smallest change (Figure 6).

**Figure 7:**
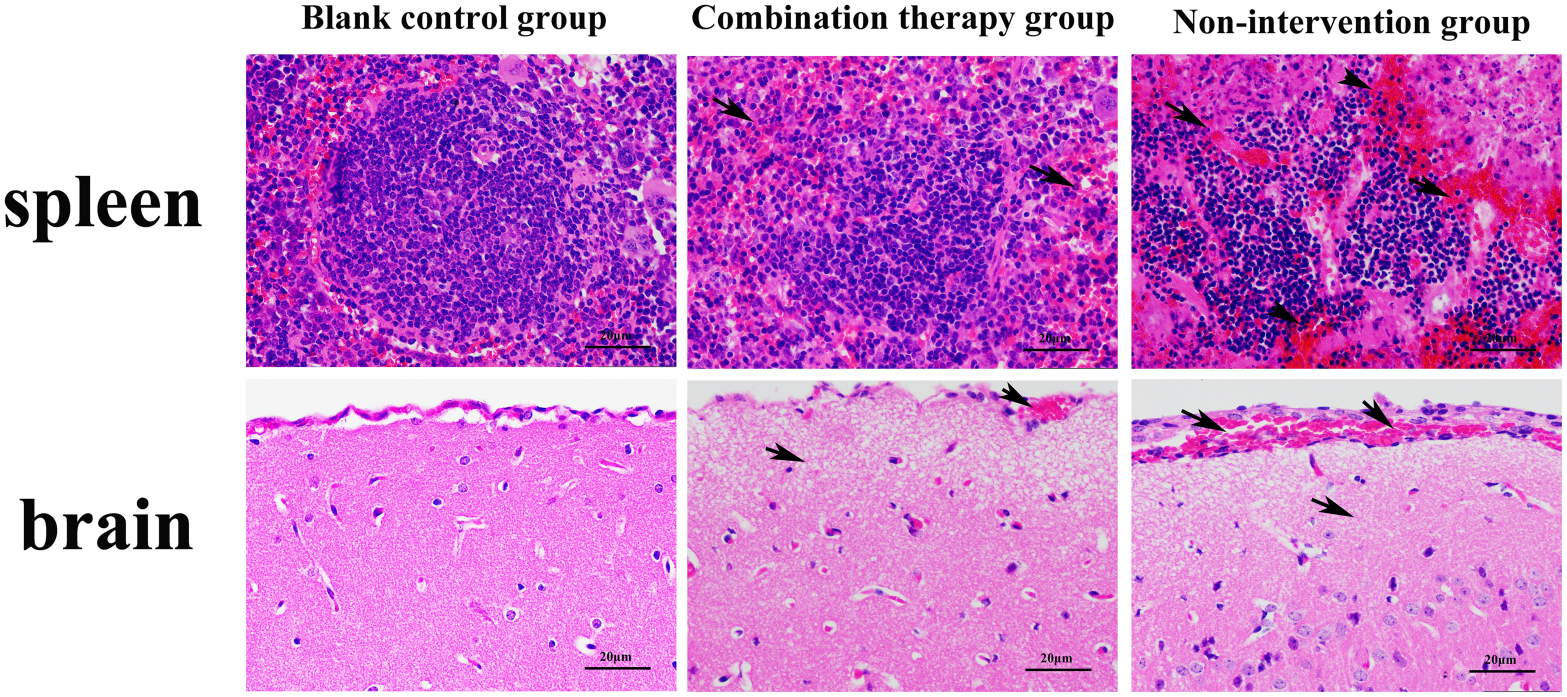
Giemsa staining of mouse parenchyma (×1,000). Red arrow indicates bacteria with the typical form of LM. LM was not detected in the blank control group and non-intervention group.

**Figure 8:**
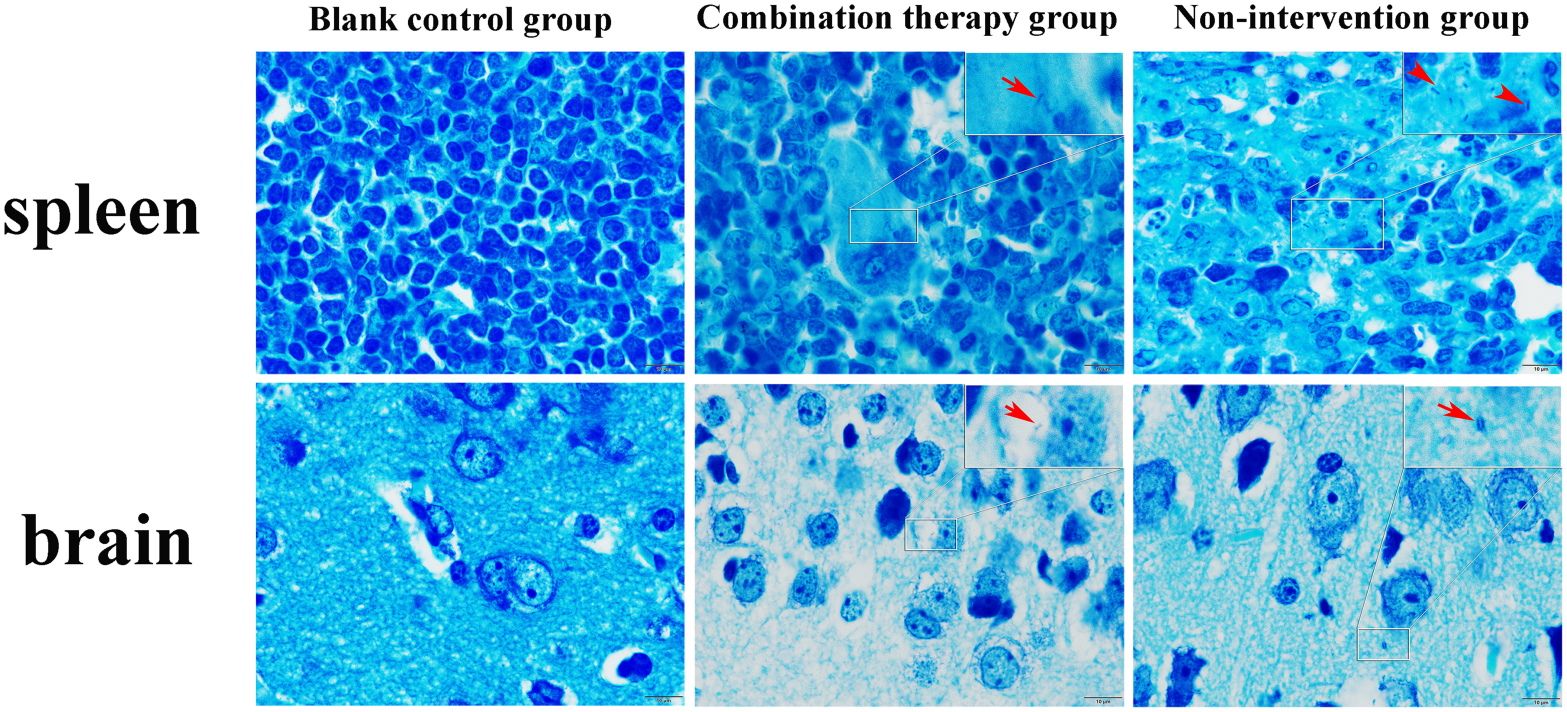
Spleen bacterial load in the listeriosis model. **(A)** Blank control group. **(B)**. Toxic reaction group. **(C)** Penicillin G sodium treatment group. **(D)** Phage Therapy Group. **(E)** Combination Therapy Group. **(F)** Non-intervention group. The values are means and standard deviations (SD) (*n* = 5). *C < 0.05. Each group vs. combination therapy group. *N < 0.05. Each group vs. non-intervention group.

For pathological changes, the results for the no-intervention group showed that LM caused severe hemorrhagic necrosis in the spleen, meningeal local hemorrhage, hemorrhagic necrosis and atrophy of glomeruli, diffuse hemorrhage of the liver accompanied by severe edema, and atrophy and rupture of the cardiac myocardial fibers (Figure 7). In the late stage, symptoms such as secondary bacteremia and meningitis appeared, and the kidneys, heart, and liver displayed various degrees of atrophy (Appendix 8). Bacteria with the typical morphology of LM were found in the parenchymal organs, especially in the spleen and brain (Figure 8).

**Figure 9:**
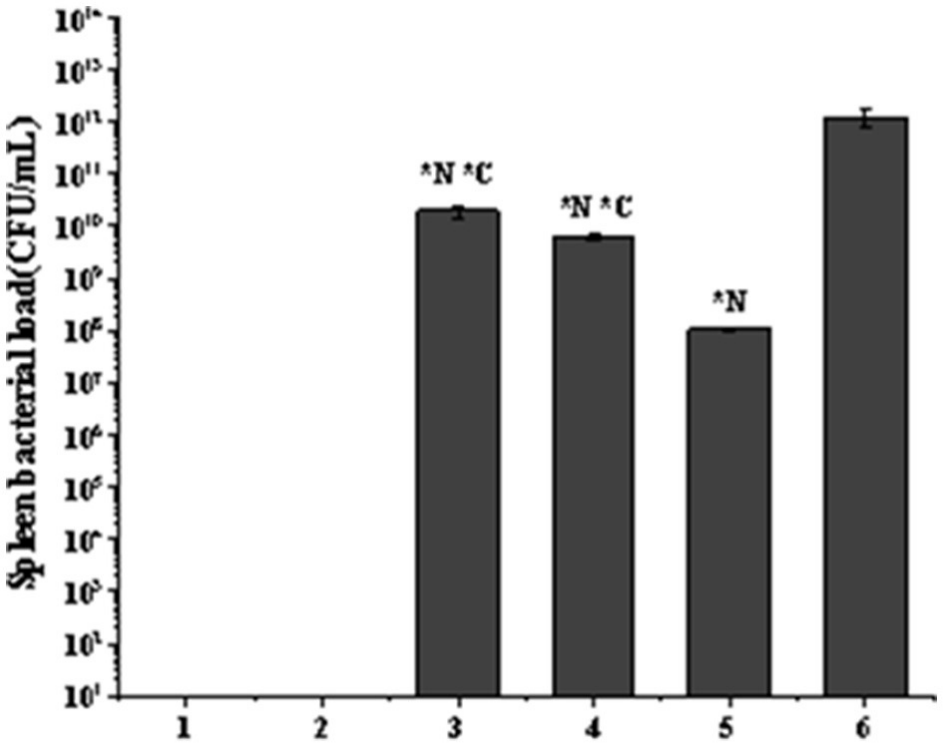
Hematoxylin–eosin staining microscopy of spleen and brain (×400). Black arrow in the figure indicates the location of the lesion. Compared with the blank control group, the non-intervention group showed partial splenic corpuscle disappearance from spleen; apoptosis of a large number of lymphocytes, accompanied by a certain degree of hemorrhage, and the boundary of the germinal center was blurred. Partial meningeal hyperemia, cerebral cortex tissue shows watery degeneration, and the nerve cells are necrotic and atrophied, which is typical of sepsis and meningitis.

There was no bacterial growth on the plates in the blank control group or the toxic reaction group. The bacterial load was highest in the no-intervention group with 1.23 × 10^12^ CFU/mL. The load in the phage treatment group was 6.27 × 10^9^ CFU/mL, and the antibiotic treatment group was 1.97 × 10^10^ CFU/mL. However, the combination therapy group was 1.05 × 10^8^ CFU/mL (Figure 9).

## Discussion

First, LP8 was isolated from slaughterhouse sewage contaminated with LM. Whole-genome sequencing showed that the genome is 87,038 bp. LP8 belongs to the family of *Myoviridae*. By comparing the whole genome sequence of LP8, the coding region of the phage genome was more than 80% of the genome size. Among the genes encoded, 76 genes were highly similar to some genes from *Salmonella, Escherichia*, and *Citrobacter* phages, with maximum single coverage of 7% and cumulative coverage of 38.4%. For the 120 genes, the similarity of BLAST from the NCBI is low, and the function of 44 genes is unknown. The above phenomenon indicates that LP8 may have undergone a complex process of horizontal gene transfer, mutation, and recombination during the evolution process (21, 22).

At present, the LISTEX™ P100 is used to prevent and control food safety incidents caused by LM (23). The reagents are largely based on six species of LM phage, and this cocktail therapy is used to deal with the problem of the high specificity of a single phage. Compared with LISTEX™ P100, LP8 has a shorter action time and wider temperature range for activity. It is also effective in combination with antibiotics. LP8 has a greater capacity for cross-species lysis and a shorter incubation period than other reported LM phages (24, 25).

Song et al. measured the one-step growth curve of LM phages LP020, LP027, and LP094, and the results showed that the incubation period of these three LM phages was 45∽60 min (25). However, the incubation period of LP8 was 23 min, reaching the plateau after 80 min. The four phages with the highest efficiency were all above 1 × 10^10^ PFU·mL^−1^ and had the same effect, but LP8 acted faster. Lee et al. isolated two LM phages LMP1 and LMP7 from chicken manure samples, which showed lytic activity in ATCC7644, ATCC15313, ATCC19114, and ATCC19115 (26). In addition to 18 strains of LM, LP8 also showed lytic activity against five strains of *L. welshimer* (9-2, 4-40, C10, C59, 4-43), indicating that LP8 had cross-species lytic activity.

*L. monocytogenes* is classified into four evolutionary lines (I, II, III, and IV) and four molecular serogroups (IIa, IIc, IIb, and IVb), which cover different serotypes (1/2a, 3a, 1/2c, 3c, 1/2b, 3b, 4b, 4d, and 4e) (27–29). Several studies have indicated strains divergence regarding their ability to persist in the environment, as well as their virulence potential (29), because the serotyping of *L. monocytogenes* is based on the difference between the A antigenic site and the O antigenic site on the surface of the bacteria. LP8 can lyse 18 strains of *L. monocytogenes*, among which the lysis ability is generally higher for the strains with serotype 1/2a; therefore, we suspect that the recognition of *L. monocytogenes* by LP8 is related to the A and O antigens on the bacterial surface. In addition, LP8 has a lytic activity to five strains of *L. welshimeri*. According to our evolutionary analysis of the 16s RNA of 10 strains of *L. welshimeri*, the five strains that LP8 can lyse with are located on the same evolutionary branch. LP8 had no lytic activity against 22 strains of *L. innocua*, which may be related to the different surface sites of the bacteria.

The results of the three treatment groups in this study showed that the lymphocyte subsets decreased, and the expression of cytokines increased, proving that the three methods had an intervention effect on LM infection. Because of their biological activity and composition, phages are susceptible to complex environments, so combining them with antibiotics can compensate for some shortcomings of the phages themselves (30, 31). In addition, the effect of the combination group was better than that of the single administration group, indicating that the addition of phage can reduce the use of antibiotics. In the future, genetically modified phages could specifically replace resistant genes in bacterial genomes, making bacteria susceptible to antibiotics again (32). This also demonstrates the potential of phage-antibiotic combinations as novel biologics.

## Conclusion

This study confirms that LP8, an LM phage isolated from slaughterhouse sewage, was effective for LM and *L. welshimeri*. The combination of LP8 and antibiotics can compensate for the impact of the biological activity of the phage in the treatment. Furthermore, the addition of phage can reduce the use of antibiotics and achieve the effect of reducing and limiting antibiotics finally.

## Data Availability Statement

The datasets presented in this study can be found in online repositories. The names of the repository/repositories and accession number(s) can be found below: https://www.ncbi.nlm.nih.gov/genbank/, OK283618.1.

## Ethics Statement

The animal study was reviewed and approved by Animal Welfare and Ethics Committee of Gansu Agricultural University.

## Author Contributions

This article is provided by HG and HX to provide design ideas, guide the experimental process, follow-up data analysis and paper writing. TL is engaged in related experimental work, data analysis and paper writing. XW and ZW provide relevant experimental sample collection work. XZ and CT assisted in phage isolation. WS and YQ assisted in phage identification. HW and CS assisted in mouse experiments. All authors contributed to the article and approved the submitted version.

## Funding

This work was supported by the National Nature Science Foundation of China (nos. 32060822, 31960726, and 31560700), Key Research and Development Program of Gansu Province (no. 20YF8-FA136), National Key Research and Development Program of China (no. 2019YFC1605705), Gansu Agricultural University Youth Tutor Support Fund (no. GAU-QDFC-2020-10), and Gansu Excellent Postgraduate Innovation Star Project (no. 2021CXZX-390). We are indebted to international science editing for the critical correction of this manuscript.

## Conflict of Interest

The authors declare that the research was conducted in the absence of any commercial or financial relationships that could be construed as a potential conflict of interest.

## Publisher's Note

All claims expressed in this article are solely those of the authors and do not necessarily represent those of their affiliated organizations, or those of the publisher, the editors and the reviewers. Any product that may be evaluated in this article, or claim that may be made by its manufacturer, is not guaranteed or endorsed by the publisher.
