# Supplementary material for: Characterization and Preliminary Application of Phage Isolated From Listeria monocytogenes
Source: Front Vet Sci. 2022 Jul 20;9:946814. doi: 10.3389/fvets.2022.946814 (PMC9387353; doi:10.3389/fvets.2022.946814)
Supplement: Supplementary file 1 [file Data_Sheet_1.docx]

**Appendix 1 Test grouping information**

| Test group | Specific allocation | Usage meaning |
| --- | --- | --- |
| Blank control group | Injected with 100 μL of SM buffer, without any further treatment | Blank control group |
| Toxicity response group | Injected witht 100 μL of phage fluid | Detection of phage toxicity |
| Antibiotic treatment group | After challenge, treated with antibiotics only | To evaluate the effect of antibiotics on the treatment of listeriosis |
| Phage treatment group | After challenge, treated with phage only | To evaluate the effect of bacteriophage on the treatment of listeriosis. |
| Combination therapy group | After challenge, treated with a phage–antibiotic mixture | To evaluate the feasibility of using bacteriophage to reduce the use of antibiotics. |
| Modeling nonintervention group | Each mouse was injected intraperitoneally with 100 μL of *Listeria monocytogenes* suspension | The single bacterial infection group did not receive any treatment and was used as the follow-up control |

**Appendix 2 The measured conversion relationship of ATCC19111 that between the bacterial concentration and the OD value**

**Appendix 3 Minimum Inhibitory Dosage Determination of Six Commonly Used**

**Antibiotics for Listeriosis**

| Types of antibiotics | Documentation MIC（μg/L） | Test and measurement  MIC（μg/L） | selection criteria |
| --- | --- | --- | --- |
| Penicillin G sodium | 16~＜128 | 11, 000 | Wide range of applications, easy to operate, and insensitive to strains |
| cefotaxime | unknown | ＞100 | Inconvenient to operate, the strain is more sensitive |
| Sulfamethoxazole | unknown | 40 | The strain is very sensitive, and the effect of combination with bacteriophage is not obvious |
| tetracycline | 32~＜64 | 10 |  |
| erythromycin | 8~＜32 | 60 |  |
| Gentamicin | 32 | 10 |  |

**Appendix 4 Plate of single penicillin G sodium antibacterial test**


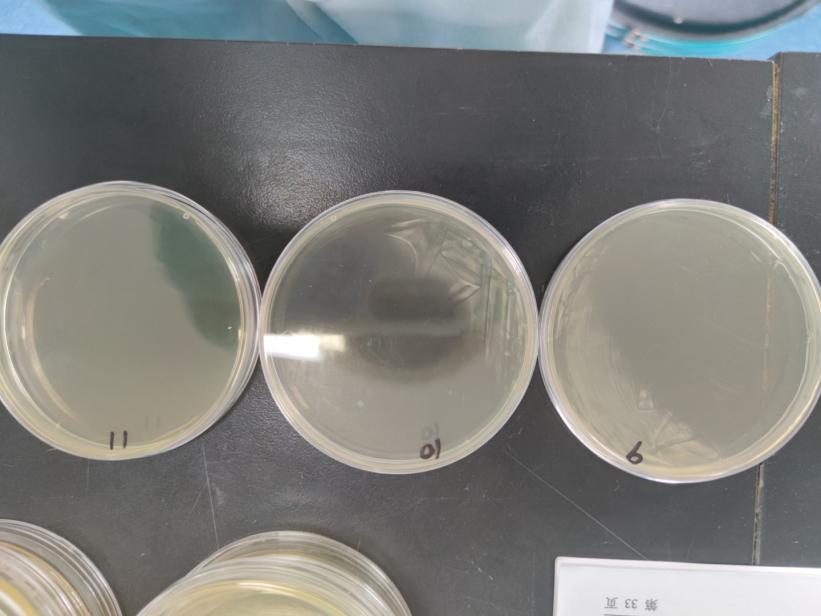


Note:The numbers marked on the plates in the figure represent different concentrations of penicillin G sodium, which are 9 mg/L, 10 mg/L and 11 mg/L

**Appendix 5 plate of penicillin G sodium combined with LP8 antibacterial test**


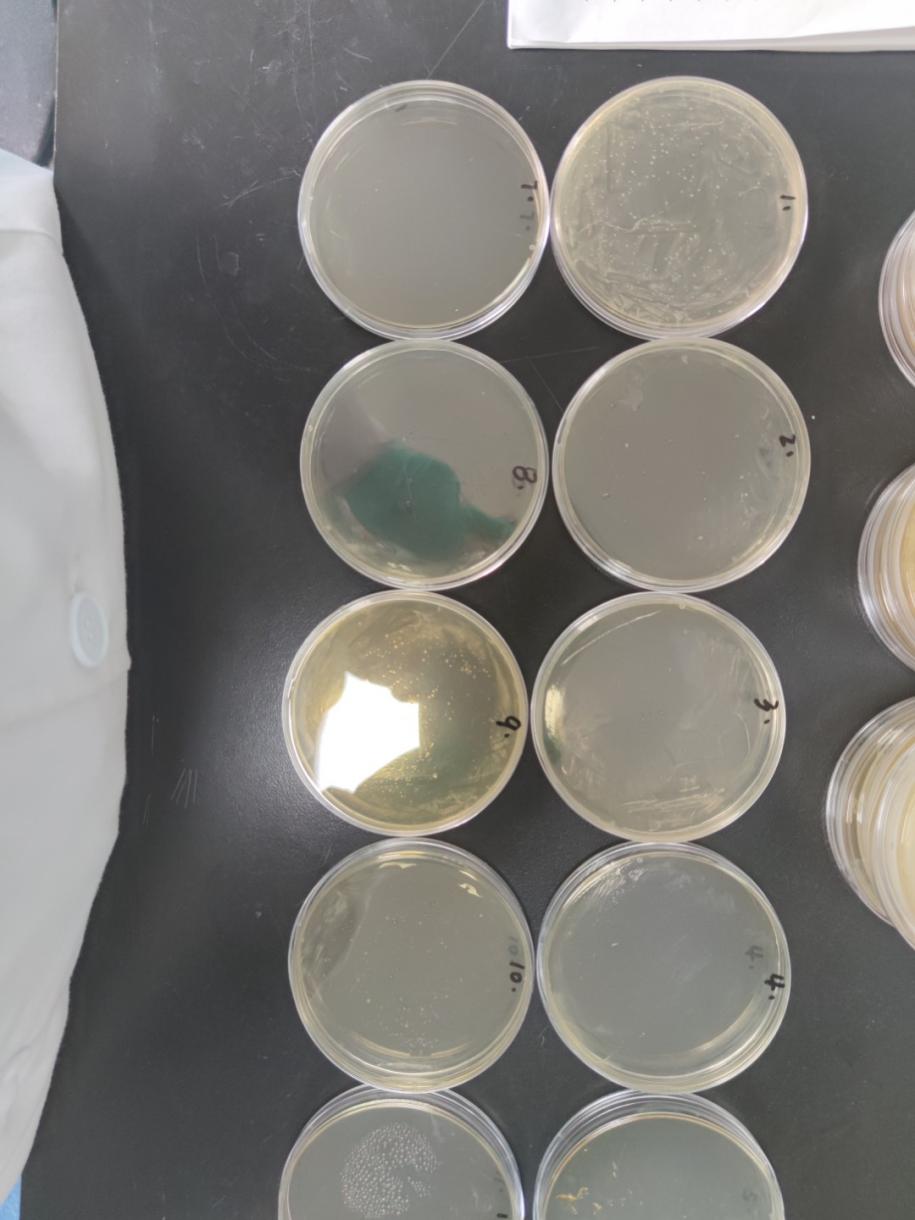


Note:The concentration of LP8 is 2.09×10^10^ PFU/mL. The numbers marked on the plate in the figure represent different concentrations of penicillin G sodium, which are 1 mg/L, 2 mg/L, 7 mg/L and 8 mg/L

**Appendix 6 The host spectrum of LP8**

| Serial number | Bacteria Strains | Bacteria number | Bacteria Serotype | Electroplating efficiency | LP8 cleavage |
| --- | --- | --- | --- | --- | --- |
| 1 | *L.monocytohenes* | D46-1-1 | 1/2a | 0.897 | + |
| 2 |  | C64-2 | 1/2c | 0.81 | + |
| 3 |  | A77-1-2 | 1/2a | 0.983 | + |
| 4 |  | A78-2-2 | 1/2b | 0.88 | + |
| 5 |  | A83-2-1 | 1/2c | 0.88 | + |
| 6 |  | D31-1 | 1/2a | 0.897 | + |
| 7 |  | 2-20-1 | 4b | 0.828 | + |
| 8 |  | C18-2-1 | 1/2c | 0.914 | + |
| 9 |  | C33-1-2 | 1/2a | 0.93 | + |
| 10 |  | A85-1-1 | 1/2b | 0.845 | + |
| 11 |  | A79-1 | 1/2a | 0.897 | + |
| 12 |  | 1-12-2-2 | 1/2c | 0.74 | + |
| 13 |  | 5-90-1-2 | 1/2c | 0.71 | + |
| 14 |  | 6-56-1-2 | 1/2c | 0.759 | + |
| 15 |  | ATCC19112 | 1/2c | 0.81 | + |
| 16 |  | NCTC10890 | 1/2b | 0.638 | + |
| 17 |  | ATCC19111 | 1/2a | 1 | ++ |
| 18 |  | ATCC19115 | 4b | 0.71 | + |
| 19 | *L. welshimeri* | A92 |  | 0 | - |
| 20 |  | 4-27 |  | 0 | - |
| 21 |  | C84 |  | 0.21 | - |
| 22 |  | D27 |  | 0 | - |
| 23 |  | 1-95 |  | 0 | - |
| 24 |  | 9-2 |  | 0.515 | + |
| 25 |  | 4-40 |  | 0.528 | + |
| 26 |  | C10 |  | 0.651 | + |
| 27 |  | C59 |  | 0.681 | + |
| 28 |  | 4-43 |  | 0.531 | + |
| 29 | *L. innocua* | 6-33 |  | 0 | - |
| 30 |  | 8-88 |  | 0 | - |
| 31 |  | 9-100 |  | 0 | - |
| 32 |  | 12-52 |  | 0 | - |
| 33 |  | 10-35 |  | 0 | - |
| 34 |  | C72 |  | 0 | - |
| 35 |  | A22 |  | 0 | - |
| 36 |  | D98 |  | 0 | - |
| 37 |  | A78 |  | 0.135 | - |
| 38 |  | C91 |  | 0.002 | - |
| 39 |  | D59 |  | 0 | - |
| 40 |  | 1-25 |  | 0 | - |
| 41 |  | B1 |  | 0.017 | - |
| 42 |  | 10 |  | 0 | - |
| 43 |  | 7 |  | 0 | - |
| 44 |  | 8-33 |  | 0 | - |
| 45 |  | 6-99 |  | 0 | - |
| 46 |  | 8-54 |  | 0 | - |
| 47 |  | 4-40 |  | 0 | - |
| 48 |  | 5-2 |  | 0 | - |
| 49 |  | 4-68 |  | 0 | - |
| 50 |  | 4-99 |  | 0 | - |

Note: +++，Efficiency of plating (EOP) of 2–1.5；++，EOP of 1.5–1；+，EOP of 1– 0.5；—，No cleavage of phage


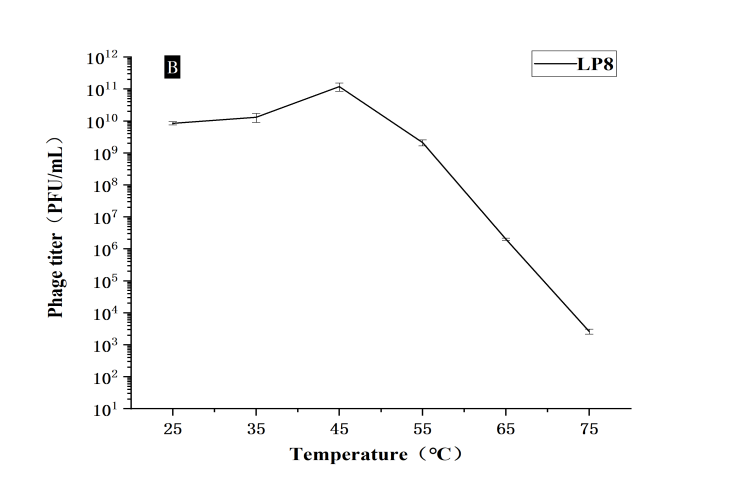

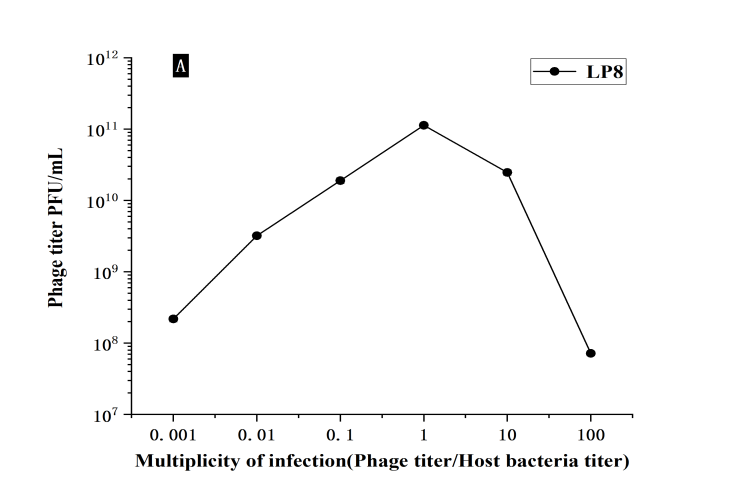


**Appendix 5**


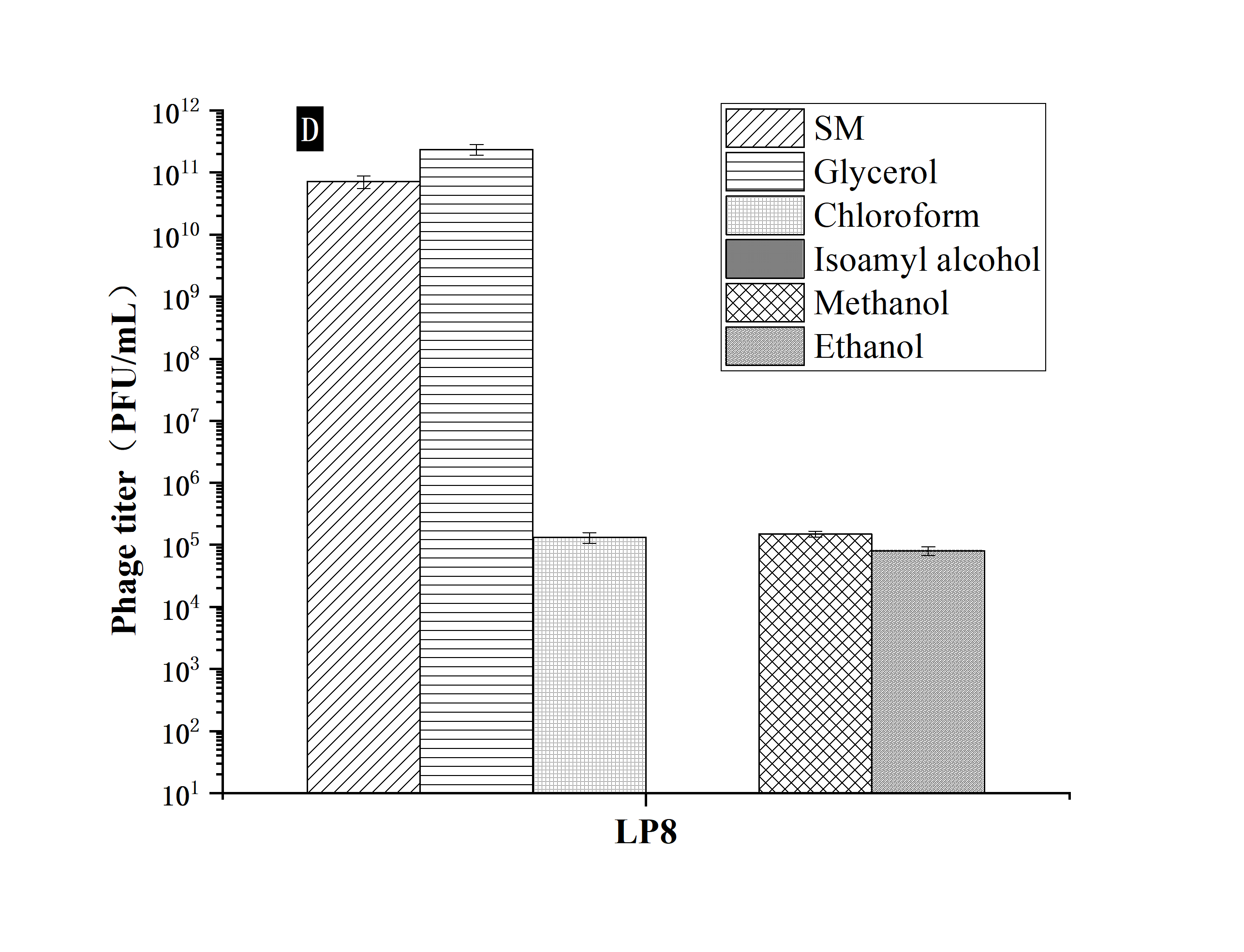


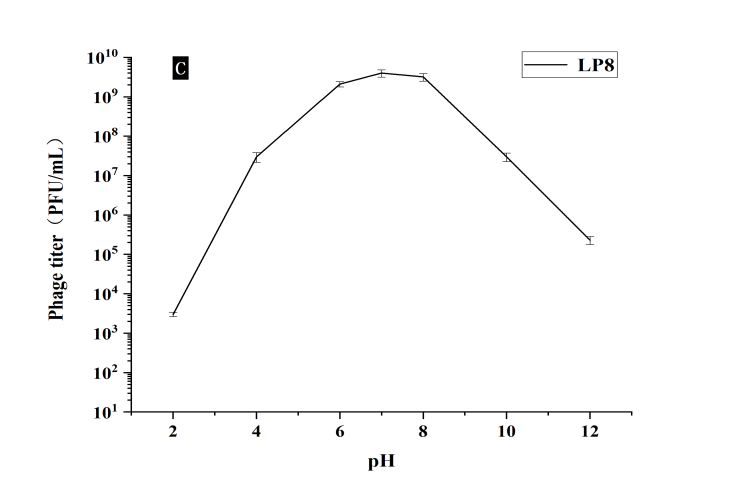


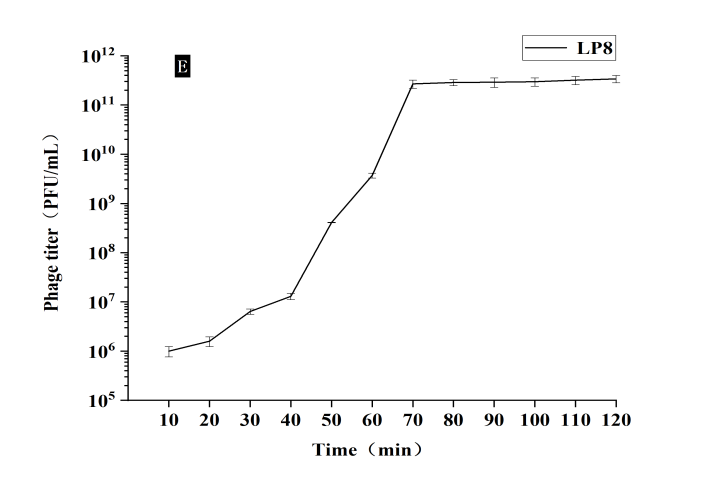


**Appendix7 Four Growth characteristics of phage LP8**

A： optimal multiplicity of infection； B：temperature influence；C：pH influence； D： organic solvent influence；E： one-step growth curve**;**


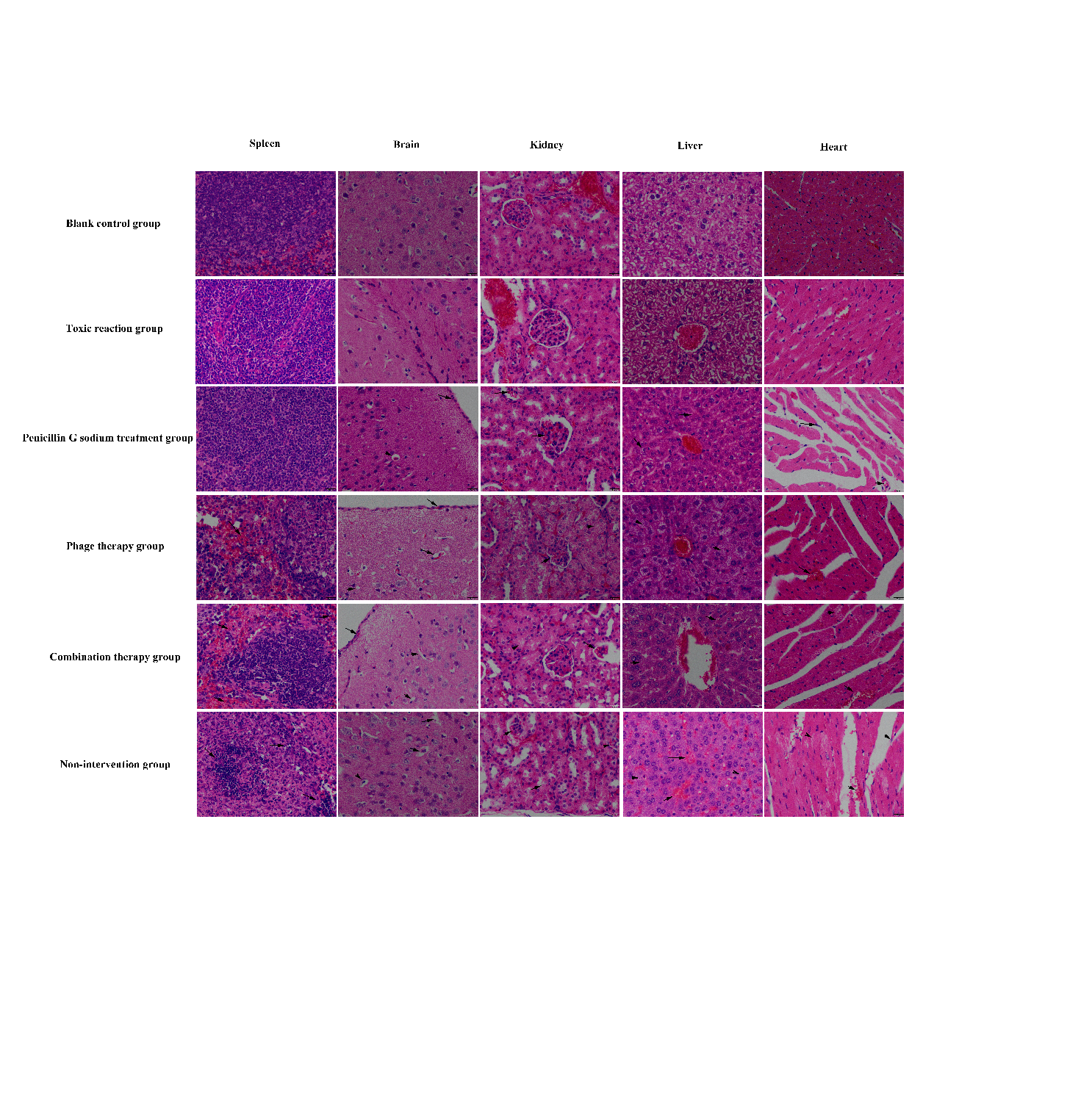


**Appendix 8 Hematoxylin–eosin staining of mouse organs, ×400**
